# Intercalation of calcein into layered silicate magadiite and their optical properties

**DOI:** 10.1098/rsos.171258

**Published:** 2017-11-29

**Authors:** Shengnan Gao, Jiang Zhu, Yifu Zhang, Qiushi Wang, Xuyang Jing, Changgong Meng

**Affiliations:** 1 School of Chemistry and Chemical Engineering, Liaoning Normal University, Dalian 116029, People's Republic of China; 2 School of Chemistry, Dalian University of Technology, Dalian 116024, People's Republic of China

**Keywords:** layered silicates, magadiite, calcein, solid-state intercalation, fluorescence

## Abstract

Calcein–Ca (II), Zn (II) and Al (III) complexes were successfully intercalated into interlayer surfaces of layered silicate magadiite and fluorescence properties of organic metal-chelates in the confined spaces were investigated. Structures, compositions and morphologies of the intercalated magadiites were adequately studied by tests, including X-ray diffraction, energy-dispersive X-ray spectrometer, elemental mapping, X-ray photoelectron spectroscopy, inductively coupled plasma atomic emission spectroscopy, Fourier-transform infrared spectra, ultraviolet–visible spectroscopy, thermo-gravimetric analysis, differential thermal analysis and scanning electron microscopy. Results confirmed that metal–organic chelate species were immobilized onto the silicate sheets via solid-state interaction. Basal spacings between silicate layers decreased by exchanged metal ions and increased after intercalation of calcein into the interlayer spaces of cation-exchanged magadiites. The encapsulation was obtained by a flexible solid–solid reaction, and the present reaction and products had a potential of application to industrial uses. A speculative mechanism was proposed for reaction by solid-state intercalation. Furthermore, it was found that the complexes in the interlayer space also exhibited special fluorescence properties. The significance of this current work was that it provided a possible route for synthesizing metal–organic complexes that encapsulated in phyllosilicate.

## 1. Introduction

**Scheme 1.**
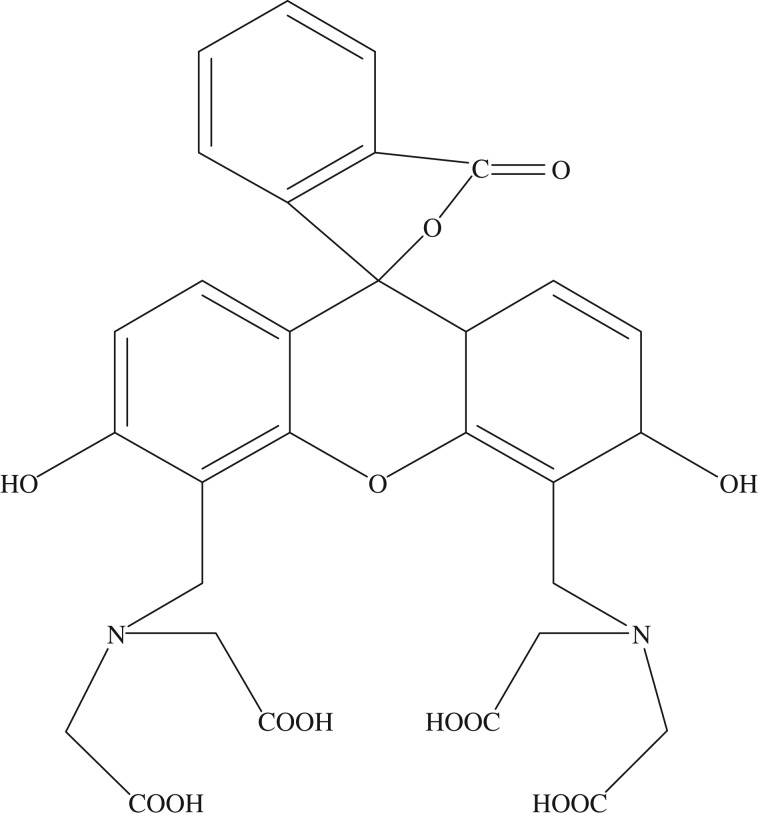
Molecular structure of calcein.

Intercalation of guest species into layered inorganic materials has attracted great attention from a wide range of scientific and practical viewpoints [1–5]. Intercalation of photoactive species like organic dyes into layered materials has been investigated to understand the nature of host–guest systems and to prepare novel photofunctional supramolecular systems [6], because the characteristics of the photoprocesses are sensitive to the environment in which the photoactive species are located [7]. Layered silicates, whose frameworks comprised SiO_4_ tetrahedra, included interlayer exchangeable cations which were often hydrated. As a member in the family of layered silicates, magadiite (Na_2_Si_14_O_29_·*x*H_2_O), had a layered appearance consisting of agglomerated particles with irregular shapes and partly sharp grain boundaries. The structure of magadiite was composed of multiple negatively charged sheets of SiO_4_ tetrahedra with abundant silanol-terminated surfaces. Negative charges in layers of magadiite were counterbalanced by hydrated cations (e.g. Na^+^, H^+^) in its interlayer spaces [8–10]. Magadiite had a high cation exchange capacity (CEC) which was applied to ion-exchange, whereby sodium ions could be replaced by protons, other cations or large quaternary ammonium ions, therefore it was proven to be a good candidate for fabrication of organic–inorganic composites [6,10–15]. Compared with other layered silicates like kenyaite, makatite, etc. and clay like montmorillonite, saponite, etc. magadiite which was used as host material to fabricate organic–inorganic compounds had less been reported. Calcein, a familiar fluorescent reagent, was widely used in fluorescence analysis to determine many metal ions and as an indicator for titration analysis [16]. The molecular structure of calcein is shown in scheme 1. To the best of our knowledge, the intercalation of calcein into cation-exchanged magadiite has not yet been reported.

Herein, we report the intercalation of calcein into the interlayer spaces of cation-exchanged magadiites. Calcein and its substituted complexes (denoted as metal–calcein) had attracted considerable attention owing to their practical luminescence properties [17–20]. Since the optical properties were influenced by crystal structures and molecular packings, the immobilization of metal–calcein in solid matrices may affect the light-emitting properties [21,22]. Therefore, the intercalation of metal–calcein into solid matrices is worth investigation. In the present work, solid–solid reactions between Ca-, Zn-, Al-magadiites and calcein were carried out to self-assemble metal–calcein complexes in the interlayer spaces of magadiite. Composition, structure, morphology and optical properties of intercalated magadiites were thoroughly studied.

## 2. Experimental section

### 2.1. Materials and methods

All chemicals with analytical grade, including sodium hydroxide (NaOH), zinc acetate dihydrate (C_4_H_6_O_4_Zn·2H_2_O), calcium chloride (CaCl_2_), aluminium sulfate (Al_2_(SO_4_)_3_·18H_2_O), calcein (C_30_H_26_N_2_O_13_) and silica gel (40 wt%, Aldrich), were purchased from Sinopharm Chemical Reagent Co., Ltd and used without any further purification. Initial magadiite was hydrothermally synthesized based on our previous report [23]. In a typical synthesis, mixtures of colloidal silica (Ludox) and NaOH with a molar ratio SiO_2_ : NaOH : H_2_O = 9 : 3 : 162 were sealed in a Teflon-lined autoclave and hydrothermally treated at 150°C for 48 h. After reaction, the suspension was filtered and washed carefully with distilled water to remove excess NaOH, and dried at 80°C for 24 h. The empirical chemical formula of the obtained magadiite can be expressed as Na_2_Si_14_O_29_·9H_2_O. CEC of magadiite was 200 meq/100 g [24].

### 2.2. Preparation of the intercalation of calcein into the interlayer spaces of cation-exchanged magadiites

**Scheme 2.**
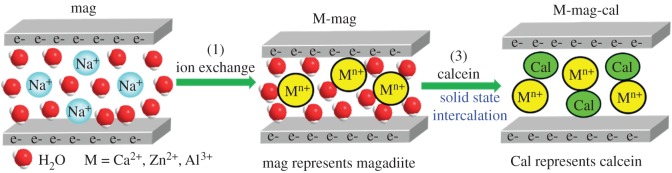
A schematic illustration of synthesis of intercalation of calcein into the interlayer spaces of Ca-, Zn-, Al-magadiites (Ca-magadiite–calcein, Zn-magadiite–calcein and Al-magadiite–calcein).

Synthesis of intercalation of calcein into the interlayer spaces of cation-exchanged magadiites is mainly composed of two steps, as depicted in scheme 2.

#### 2.2.1. Synthesis of cation-exchanged magadiites

Metal ions (Ca^2+^, Zn^2+^ and Al^3+^) exchanged magadiites (denoted as M-magadiite) were synthesized by an ion-exchange method. One molar fresh aqueous solution of Ca^2+^, Zn^2+^ or Al^3+^ was separately mixed with magadiite and these mixtures were magnetically stirred at room temperature. After 24 h, resulting products were obtained with a centrifugal separator and washed with deionized water several times until a negative AgNO_3_ test was achieved. It was noted that during synthesis of Zn^2+^ and Al^3+^ exchanged magadiite we used HCl (0.1 M) to adjust pH to prevent their hydrolysis. Obtained samples were named Ca-magadiite, Zn-magadiite and Al-magadiite, respectively.

#### 2.2.2. Solid-state intercalation of calcein into the interlayer spaces of cation-exchanged magadiites

Intercalation of calcein into the interlayer spaces of cation-exchanged magadiites was synthesized by solid–solid reactions based on the previous report [23]. Calcein was mixed with cation-exchanged magadiites and ground manually using an agate mortar and a pestle at ambient environment for 15 min. Molar ratios of calcein to interlayer cations were 2 : 1, 2 : 1, 3 : 1 for Ca-, Zn-, Al-magadiites. After solid–solid reaction, intercalated compounds were washed with ethanol several times and dried at 60°C for 24 h. Synthesized products were marked as Ca-magadiite–calcein, Zn-magadiite–calcein and Al-magadiite–calcein, respectively.

### 2.3. Materials characterization

Powder X-ray diffraction (XRD) was collected on a Panalytical X'Pert Powder diffractometer using monochromatic Cu K*α* radiation. The amounts of exchange cations were determined by inductively coupled plasma atomic emission spectroscopy (ICP-AES, PerkinElmer Optima 2000DV ICP-OES) and an energy-dispersive X-ray spectrometer (EDS) attached to a scanning electron microscope (SEM, QUANTA450). X-ray photoelectron spectroscopy (XPS) was used to investigate the surface composition of the products performed on ESCALAB250Xi, Thermo Fisher Scientific. Fourier-transform infrared (FTIR) spectra of the samples were recorded by KBr disk method on a Niole Avatar 360 FTIR spectrometer (USA) over the spectral region of 400–4000 cm^−1^. Thermo-gravimetric analysis and differential thermal analysis (TG/DTA) were taken on a Mettler-Toledo TG/SDTA-851e instrument at a heating rate of 4°C min^−1^ under a dry nitrogen atmosphere using α-Al_2_O_3_ as a standard material. TG/DTA-MS was recorded by a TG/DTA-MS combination with a Mettler-Toledo TG/DTA 1 and a Pfeiffer Vacuum Online mass spectrum analyser with QMA200M analyser and C-SEM Faraday detector. The morphology and dimensions of the products were observed by field-emission scanning electron microscopy (SEM, NOVA NanoSEM 450, FEI). Samples for SEM observation were gold-sputtered in order to get better morphology of the surface. Diffuse reflectance spectra (UV–vis) of the solid samples were collected on an American HP-8453 scanning spectrophotometer using an integrated sphere. Photoluminescence spectra (PL) were characterized on a standard Jasco FP-6500 spectrofluorophotometer with the excitation at 400 nm.

## 3. Results and discussion

**Figure 1.**
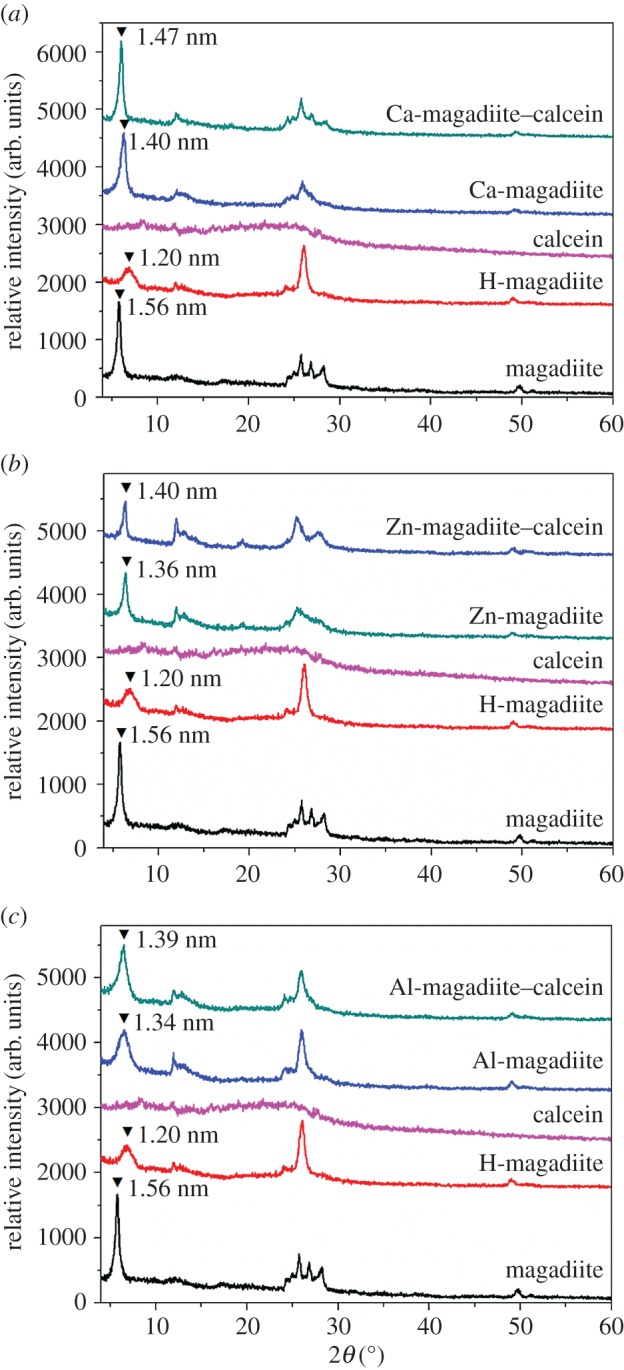
(*a*–*c*) XRD patterns of magadiite, H-magadiite, calcein, Ca, Zn, Al ion-exchanged magadiites and their intercalated compounds.

Scheme 2 displays the proposed process of intercalation of calcein into the interlayer spaces of cation-exchanged magadiites by solid–solid reactions. This route mainly consisted of ion-exchange and solid-state intercalation. Figure 1 shows XRD patterns of magadiite, H-magadiite, calcein, Ca, Zn, Al ion-exchanged magadiites and their intercalated compounds. Magadiite was successfully synthesized based on our previous report [23] and its basal spacing was 1.56 nm. As for synthesis of Ca-magadiite–calcein (figure 1*a*), Ca-magadiite was synthesized using magadiite and Ca^2+^ without adjusting pH, and XRD pattern of Ca-magadiite was similar with magadiite. Compared with magadiite, the basal spacing of Ca-magadiite decreased to 1.40 nm. After intercalation of calcein into Ca-magadiite (Ca-magadiite–calcein), the basal spacing increased to 1.47 nm. As for synthesis of Zn-magadiite–calcein (figure 1*b*) and Al-magadiite–calcein (figure 1*c*), Zn-magadiite or Al-magadiite were prepared using magadiites and Zn^2+^ or Al^3+^ with HCl adjusting pH to avoid their hydrolysis. Correspondingly, XRD patterns of Zn-magadiite and Al-magadiite were similar with types of H-magadiite. The basal spacing of Zn-magadiite and Al-magadiite measured 1.36 and 1.34 nm, respectively. Intercalation of calcein into Zn-magadiite or Al-magadiite, their basal spacing (Zn-magadiite–calcein and Al-magadiite–calcein) became 1.40 and 1.39 nm, respectively. These findings revealed that the basal spacing of cation-exchanged magadiites decreased during the ion-exchange process, which indicated that sodium counter ions were only partially replaced by the cations in the exchange reaction in regard to the original CEC of magadiite. The non-exchangeable sodium might be due to equilibrium between sodium or hydrogen and the existence of less exchangeable sites. XRD patterns of Ca-magadiite–calcein (figure 1*a*), Zn-magadiite–calcein (figure 1*b*) and Al-magadiite–calcein (figure 1*c*) showed that they had similar patterns with Ca-magadiite, Zn-magadiite and Al-magadiite, suggesting that they all had an ordered structure of clay platelets as magadiite. And their basal spacing increased after the intercalated process, suggesting the successful intercalation of calcein into the interlayer spaces of cation-exchanged magadiites [7]. Changes of the basal spacing were closely related to the peculiar arrangements of metal-chelates between the layers, and the reduced interlayer spacing could be attributed to the ‘β-type' packing in between the silicate sheets [25]. It was considered that the interlayer exchange cations of magadiite were covered by water molecules at ambient conditions; hence, the changes in the basal spacings were caused by the intercalation of calcein through ligand displacement reactions between H_2_O and calcein molecules. Therefore, calcein was recognized to be intercalated in a monolayer arrangement owing to the increased basal spacings between the layers and the different molecule packings could be due to the different conformations in the layered structures.

**Figure 2.**
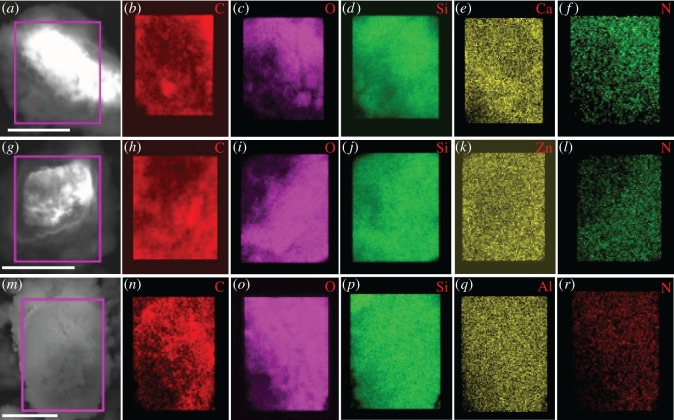
Elemental mapping images of Ca-magadiite–calcein (*a*–*f*), Zn-magadiite–calcein (*g*–*l*) and Al-magadiite–calcein (*m*–*r*).

Based on the above analyses, calcein intercalating into the interlayer spaces of cation-exchanged magadiites was achieved. To further study the composition of these magadiites, some corresponding measurements including ICP-AES, EDS, elemental mapping, XPS, FTIR and TGA were adequately carried out. Electronic supplementary material, figure S1 shows EDS spectra of Ca-magadiite–calcein, Zn-magadiite–calcein and Al-magadiite–calcein. Elements including C, N, O and Si were distinctly observed in these three samples. Correspondingly, elements Ca, Zn and Al were detected in Ca-magadiite–calcein, Zn-magadiite–calcein and Al-magadiite–calcein, respectively. Figure 2 shows elemental mapping images of Ca-magadiite–calcein, Zn-magadiite–calcein and Al-magadiite–calcein, and reveals that these three samples contained the same elements with EDS observations. From elemental mapping images (figure 2), all elements are homogeneously distributed in samples, suggesting that calcein was successfully intercalated into Ca-, Zn-, Al-magadiites.

**Figure 3.**
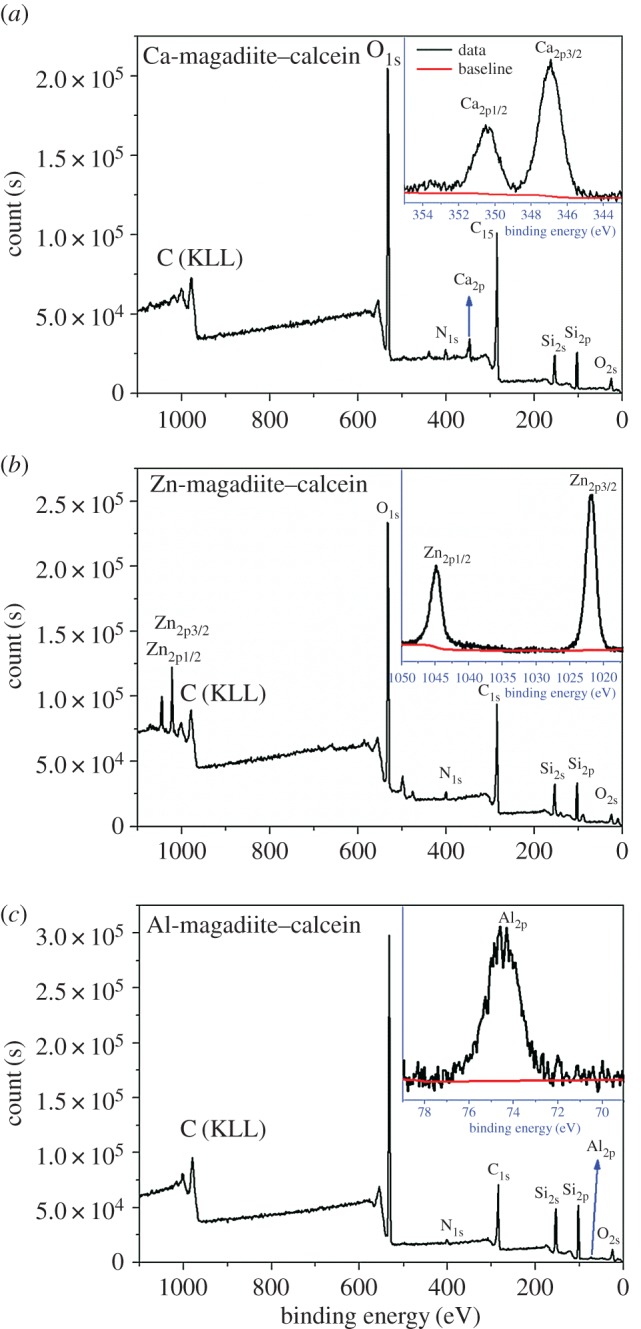
XPS spectra of Ca-magadiite–calcein (*a*), Zn-magadiite–calcein (*b*) and Al-magadiite–calcein (*c*); inserting core-level spectra of Ca_2p_, Zn_2p_, Al_2p_.

Figure 3 shows XPS spectra of Ca-magadiite–calcein, Zn-magadiite–calcein and Al-magadiite–calcein. The survey spectra revealed that these three samples all consisted of C, N, O and Si elements, and the metals Ca, Zn and Al were, respectively, observed from Ca-magadiite–calcein, Zn-magadiite–calcein and Al-magadiite–calcein. Results of XPS were well consistent with results of EDS and elemental mapping. As for Ca-magadiite–calcein (figure 3*a*), Ca_2p_ core-level spectrum split off two peaks and the binding energies at 346.8 and 350.4 eV were ascribed to Ca_2p3/2_ and Ca_2p1/2_, respectively. These data demonstrated that Ca in Ca-magadiite–calcein was +2 oxidation state. As for Zn-magadiite–calcein (figure 3*b*), Zn_2p_ core-level spectrum split off two peaks, respectively, related to Zn_2p3/2_ (1044.8 eV) and Zn_2p1/2_ (1021.7 eV) orbitals, which suggested that Zn in Zn-magadiite–calcein was +2 oxidation state. The binding energies of Ca_2p_ and Zn_2p_ differed from their corresponding oxides because the geometrical disposition of O and N coordinated with them and they were in the central place of a distorted octahedron in the phyllosilicate [26–29]. As inserted in figure 3*c*, the peak at 74.5 eV was assigned to Al_2p_. This value indicated the coordination of some N–Al or C–O–Al bonds, suggesting the existence of Al^3+^ ions in the complexes [26].

The amounts of metal cations in the intercalated magadiites were evaluated by ICP-AES test, and the results are listed in table 1. The percentage of metal was 1.03, 5.49 and 1.50 wt% of Ca, Zn and Al in Ca-magadiite–calcein, Zn-magadiite–calcein and Al-magadiite–calcein, respectively. Quantitative exchange with Ca, Zn and Al cations did not occur because of partial exchange with Na^+^ owing to the fact that different ions had different exchange abilities.

**Table 1.** Chemical content of metal in decorated magadiites.

sample	metal percentage (wt%)
Ca-magadiite–calcein	1.03
Zn-magadiite–calcein	5.49
Al-magadiite–calcein	1.50

**Figure 4.**
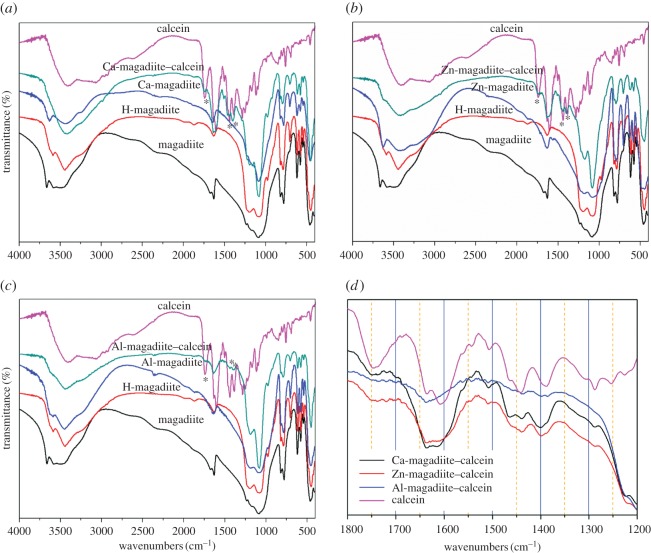
(*a*–*c*) FTIR spectra of magadiite, H-magadiite, calcein, Ca, Zn, Al ion-exchanged magadiites and their intercalated compounds; (*d*) Comparative FTIR spectra of calcein, Ca-magadiite–calcein, Zn-magadiite–calcein and Al-magadiite–calcein.

To get more information about the metal-chelates in the interlayer spacing, FTIR spectra of magadiite, H-magadiite, calcein, Ca-, Zn-, Al-exchanged magadiites and their intercalated compounds were further studied. Figure 4*a* compares FTIR spectra of magadiite, H-magadiite, Ca-magadiite, Ca-magadiite–calcein and calcein. Similar figures of Zn-magadiite–calcein and Al-magadiite–calcein are shown in figure 4*b* and figure 4*c*, respectively. From these FTIR spectra, compared with magadiite, H-magadiite and Ca-magadiite, calcein intercalated magadiites mainly show three bands originated from calcein. These three peaks are located at 1744, 1441 and 1388 cm^−1^. The band at 1744 cm^−1^ was assigned to C=O stretching band of calcein. The peak at 1441 cm^−1^ was attributed to stretching vibration of benzene ring. The wavenumber appearing at 1388 cm^−1^ was indexed to stretching vibration of benzene ring. Furthermore, figure 4*d* shows the FTIR spectra of calcein and intercalated compounds in the range of 1800–1200 cm^−1^ and table 2 summarizes the values of different peaks. The bands of intercalated products in the regions were slightly shifted when compared with those observed for calcein molecule, proving the coordination between calcein and metal interlayer cations in magadiite [7,30,31]. M–O vibration bands of intercalation compounds could not be clearly observed in samples because those of calcein complexes appeared at fingerprint region, and were overlapped by the vibration bands of magadiite [32,33]. FTIR spectra of products did not show any additional absorption bands due to decomposed species, indicating no decomposition of calcein molecule. Electronic supplementary material, figure S2 shows diffuse reflectance UV–vis absorption spectra of calcein, Ca-magadiite–calcein, Zn-magadiite–calcein and Al-magadiite–calcein. These materials exhibited similar absorption, which indicated that calcein existed in Ca-magadiite–calcein, Zn-magadiite–calcein and Al-magadiite–calcein. The above results indicated the existence of calcein between the lamellars of magadiite.

**Table 2.** Wavenumbers (cm^−1^) of FTIR band of calcein, Ca-magadiite–calcein, Zn-magadiite–calcein and Al-magadiite–calcein.

assignments	calcein	Ca-magadiite–calcein	Zn-magadiite–calcein	Al-magadiite–calcein
C=O	1744	1750	1750	1750
ring stretching	1506	1509	1508	1508
ring stretching	1441	1438	1438	1435
ring stretching	1388	1386	1386	1384

**Figure 5.**
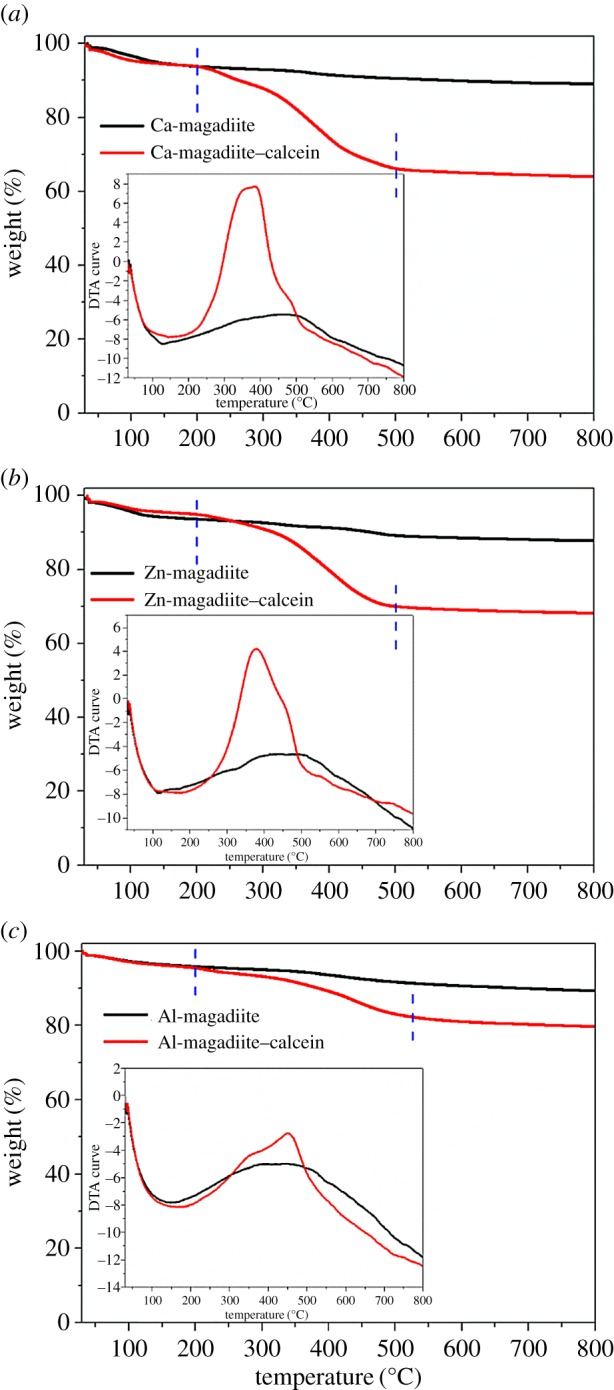
TG curves of (*a*) Ca-magadiite and Ca-magadiite–calcein, (*b*) Zn-magadiite and Zn-magadiite–calcein and (*c*) Al-magadiite and Al-magadiite–calcein; inserting their corresponding DTA curves.

To obtain the quantity of calcein and to reveal the stability of Ca-magadiite–calcein, Zn-magadiite–calcein and Al-magadiite–calcein, their corresponding TG/DTA tests were carried out, as shown in figure 5. It could be observed that cation-exchanged magadiites and their intercalated compounds mainly had three stages during the thermal degradation process. The first degradation stage before 200°C was mainly owing to the desorption of the adsorbed water in the interlayer space. The weight losses of 6.3%, 6.3%, 6.5%, 5.0%, 4.1% and 4.6% were attributed to the water molecules in the products of Ca-magadiite, Ca-magadiite–calcein, Zn-magadiite, Zn-magadiite–calcein, Al-magadiite and Al-magadiite–calcein, respectively. The second stage was in the temperature ranging from 200 to 550°C. The weight losses in this stage were 3.8%, 28.6%, 5.0%, 25.0%, 5.3% and 14.1% for Ca-magadiite, Ca-magadiite–calcein, Zn-magadiite, Zn-magadiite–calcein, Al-magadiite and Al-magadiite–calcein, respectively. The weight losses of Ca-magadiite–calcein, Zn-magadiite–calcein and Al-magadiite–calcein in this period were assigned to the decomposition of calcein, which demonstrated that calcein was contained after the intercalation. Finally, after 550°C, there was little weight loss. Therefore, it was demonstrated from weight loss variations that calcein was in Ca-magadiite–calcein, Zn-magadiite–calcein and Al-magadiite–calcein. Besides, DTA curves of Ca-magadiite–calcein, Zn-magadiite–calcein and Al-magadiite–calcein were inserted in figure 5, which revealed that the burning temperature of calcein was located at approximately 380°C. Based on all above characterizations (XRD, EDS, elemental mapping, XPS, ICP-AES, FTIR and TG/DTA), host–guest compounds including Ca-magadiite–calcein, Zn-magadiite–calcein and Al-magadiite–calcein were successfully synthesized according to our designed route.

**Figure 6.**
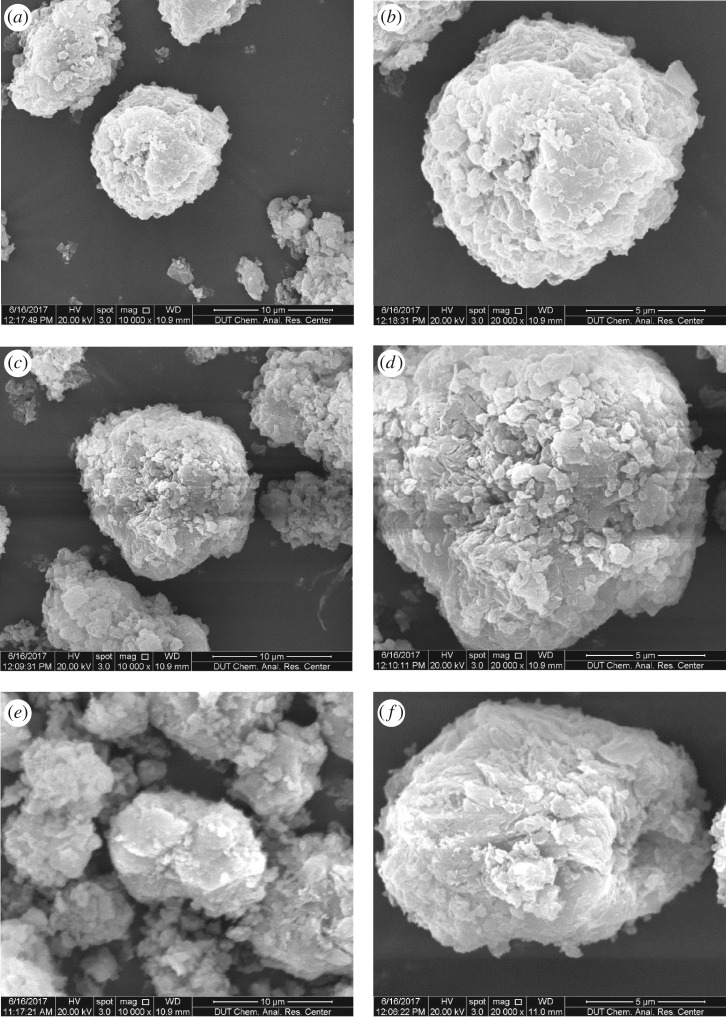
SEM images of Ca-magadiite–calcein (*a*,*b*), Zn-magadiite–calcein (*c*,*d*) and Al-magadiite–calcein (*e*,*f*).

Morphologies of magadiite, cation-exchanged magadiites and intercalated magadiites were observed by SEM. Electronic supplementary material, figures S3 and S4, and figure 6 show SEM images of magadiite, Ca-magadiite, Zn-magadiite, Al-magadiite, Ca-magadiite–calcein, Zn-magadiite–calcein and Al-magadiite–calcein, respectively. As observed in electronic supplementary material, figure S4, most of the platelets in Ca-magadiite, Zn-magadiite and Al-magadiite, were well preserved without severe destruction compared with original magadiite (electronic supplementary material, figure S3). Figure 6 depicts SEM images of Ca-magadiite–calcein, Zn-magadiite–calcein and Al-magadiite–calcein. It was observed that Ca-magadiite–calcein, Zn-magadiite–calcein and Al-magadiite–calcein kept the original platelets. However, the platelets became disordered to some extent. The morphology of platelets of various decorated magadiites was slightly destroyed, which was caused by the grinding process [23].

On the basis of the above analyses, Ca, Zn, Al ion-exchanged magadiites and intercalated magadiites were successfully prepared. The mechanism of this intercalation had not been well established, but this process might be considered as resulting from the tendency of the reaction of a neutral organic molecule and hydrated cations in the interlayer space. According to states of reactants, mechanism of calcein intercalated magadiites could be assigned to the process of solid-state intercalation. Coordination bond had been proposed as the major binding mode between calcein and ion-exchanged magadiites. Since solid–solid reactions were with high concentration, it was possible that the reactions proceed very fast. It seemed that calcein molecule had a high mobility comparable to that in the liquid state even in the solid state [34]. Soluble calcein could penetrate the water molecules to cover cations and made a coordination, which was responsible for the adsorption of various non-ionic organic compounds such as amides, alcohols, amines and ethers [35].

**Figure 7.**
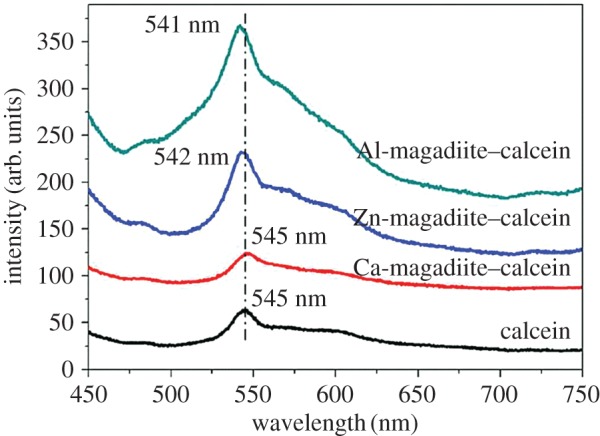
Fluorescence spectra of calcein, Ca-magadiite–calcein, Zn-magadiite–calcein and Al-magadiite–calcein.

**Figure 8.**
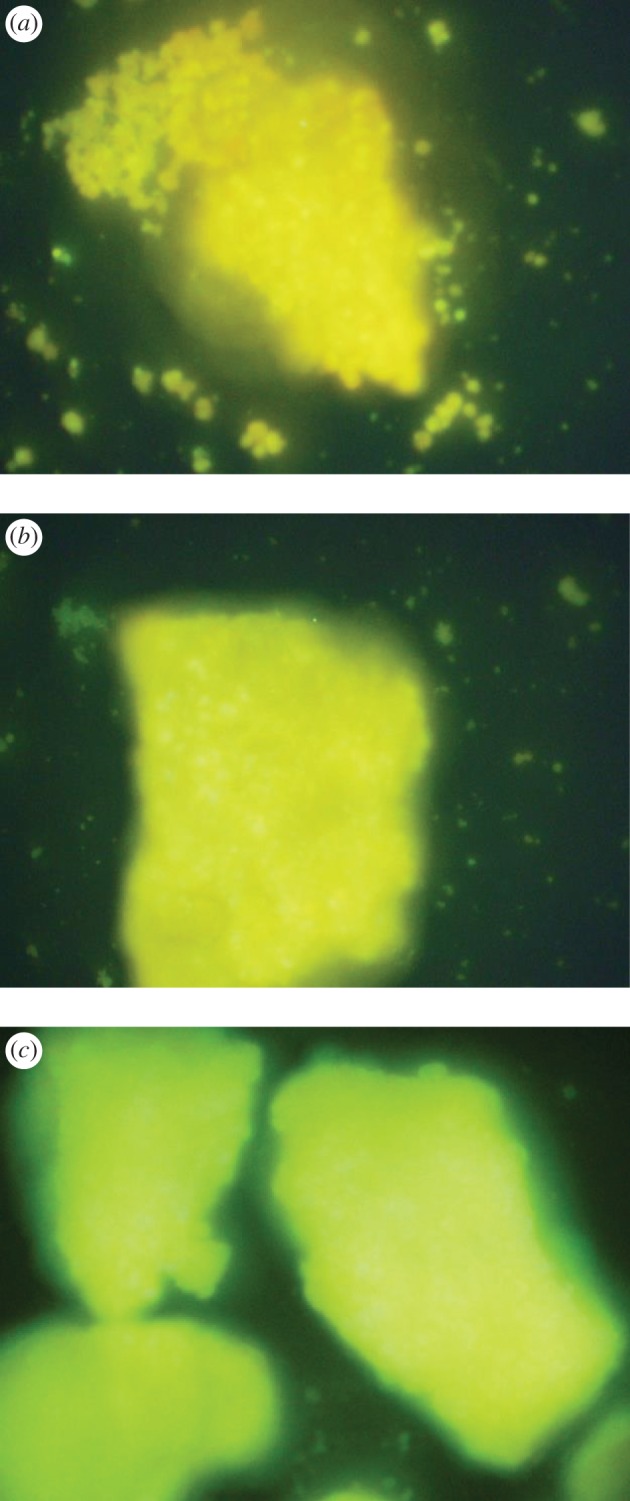
Inverted fluorescence microscope images of Ca-magadiite–calcein (*a*), Zn-magadiite–calcein (*b*) and Al-magadiite–calcein (*c*) under 250 nm UV light irradiation.

Figure 7 shows PL spectra of the intercalated compounds. In PL spectra of Ca-magadiite–calcein, Zn-magadiite–calcein and Al-magadiite–calcein, luminescence peaks appeared at around 545, 542 and 541 nm, respectively, indicating the formation of metal–calcein complexes in the interlayer space. These luminescence bands were different from the corresponding band in solution [36] because of the effect of confined region (interlayer of magadiite). Compared with calcein, the molecular structure and packing of metal–calcein formed in the interlayer of magadiite differently to make the tiny difference in the wavelength of PL spectra. Figure 8 shows photoluminescence photographs of Ca-magadiite–calcein, Zn-magadiite–calcein and Al-magadiite–calcein. As clearly demonstrated by the fluorescence microscope images, Ca-magadiite–calcein, Zn-magadiite–calcein and Al-magadiite–calcein presented yellow fluorescence, corresponding to the results from figure 7. The solid–solid reactions exhibited a successful preparation of the hybrid materials of complexes—magadiites (calcein intercalating into the interlayer spaces of cation-exchanged magadiites) that may create the opportunities to use these hybrids in designing novel optical materials.

## 4. Conclusion

In conclusion, we demonstrated that calcein was successfully introduced into the interlayer spaces of Ca-, Zn- and Al-magadiites. Metal–organic chelates species were immobilized onto the silicate sheets by solid-state interaction. Since the reaction was based on chelation between metal and organics in the interlayer space of magadiite, intercalated magadiites were proven to present luminescence properties in phyllosilicate. Basal spacings between silicate layers were controlled by exchanged metal ions and intercalated calcein, indicating the different microstructures including molecular packing of the complexes in the intercalation compounds. Owing to the easy operation of solid–solid reactions, the present reaction and product were useful for practical applications. The significance of this current work was that it provides a possible route for synthesizing metal–organic complexes that encapsulated in phyllosilicate. More studies on the preparation of intercalation compounds with controlled microstructures and functions using calcein ligand and different metal cations are in development.

## Supplementary Material

Supplementary Figures

## Data accessibility

The datasets supporting this article have been uploaded as part of the electronic supplementary material.
