# Supplementary material for: Intercalation of calcein into layered silicate magadiite and their optical properties
Source: R Soc Open Sci. 2017 Nov 29;4(11):171258. doi: 10.1098/rsos.171258 (PMC5717686; doi:10.1098/rsos.171258)
Supplement: Supplementary Figures [file rsos171258supp1.pdf]

Electronic supplementary material

Fig. S1

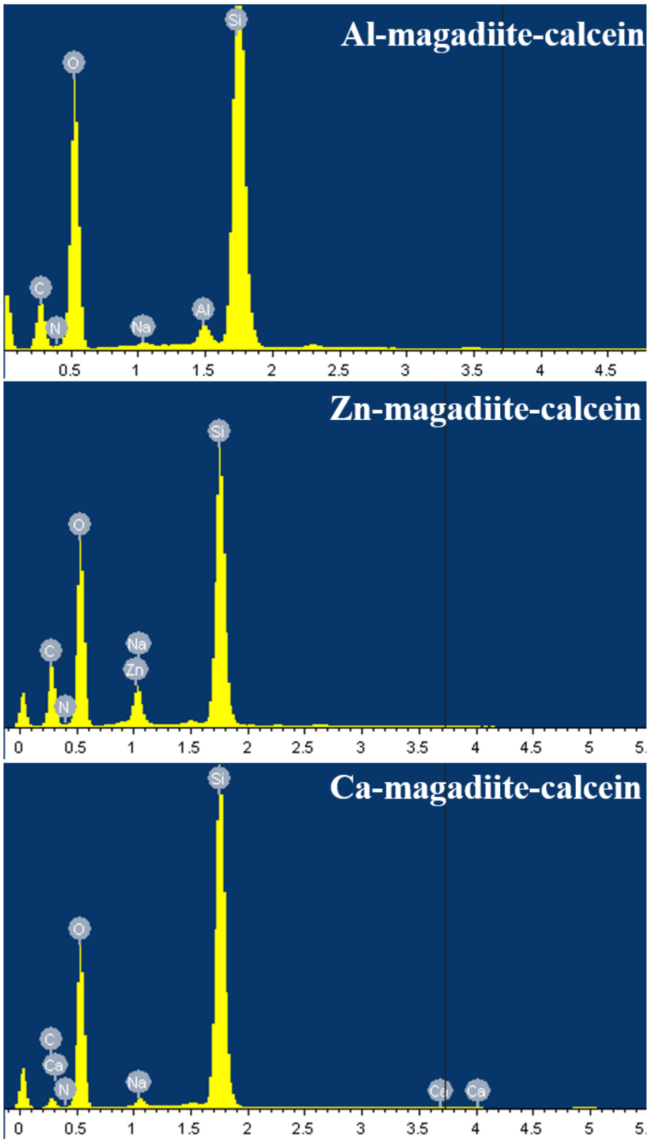

Fig. S1. EDS spectra of Ca-magadiite-calcein, Zn-magadiite-calcein and Al-magadiite-calcein.

Fig. S2

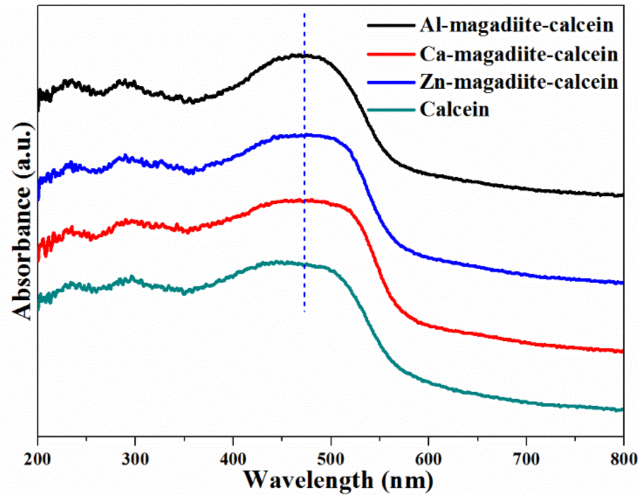

Fig. S2. Diffuse reflectance absorption spectra of calcein, Ca-magadiite-calcein, Zn-magadiite-calcein and Al-magadiite-calcein.

Fig. S3

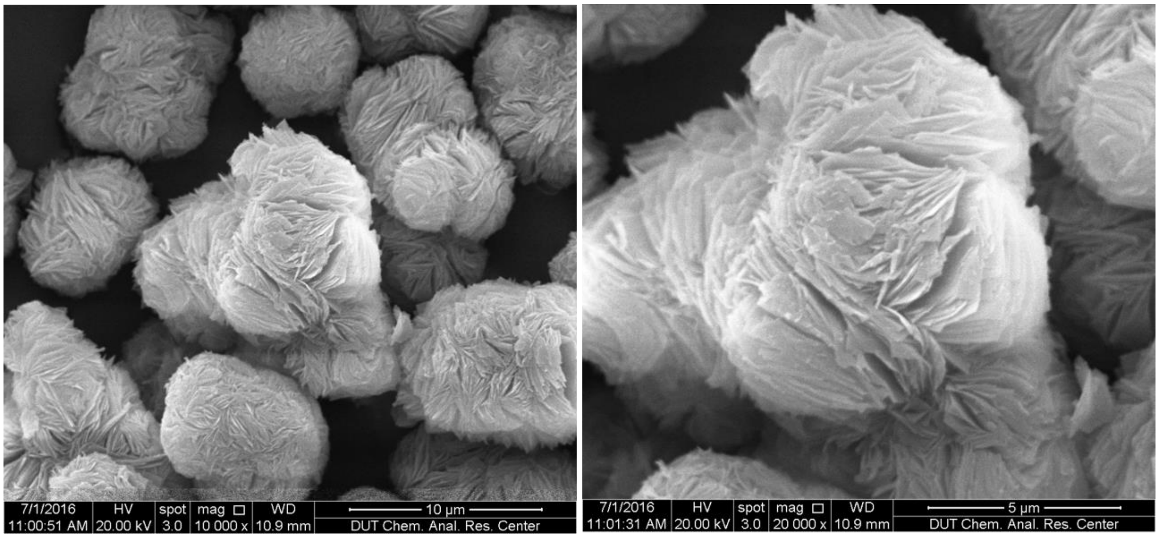

Fig. S3. SEM images of magadiite.

Fig. S4

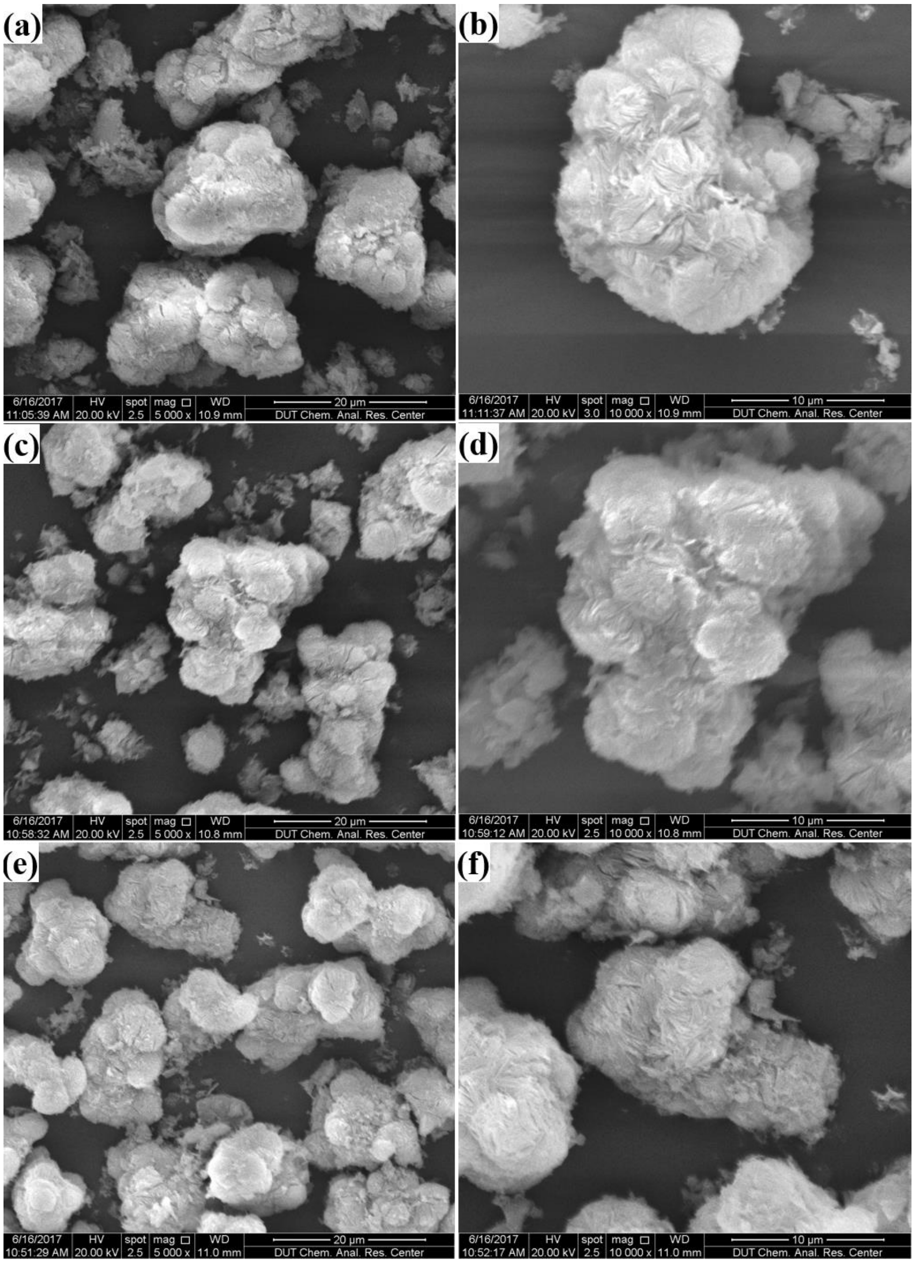

Fig. S4. SEM images of (a-b) Ca-magadiite; (c-d) Zn-magadiite, (e-f) Al-magadiite.
